# Clinical features and shared mechanisms of chronic gastritis and osteoporosis

**DOI:** 10.1038/s41598-023-31541-8

**Published:** 2023-03-27

**Authors:** Tao Han, Yili Zhang, Baoyu Qi, Ming Chen, Kai Sun, Xiaokuan Qin, Bowen Yang, He Yin, Aili Xu, Xu Wei, Liguo Zhu

**Affiliations:** 1 Department of Spine, Wangjing Hospital, China Academy of Chinese Medical Sciences, Huajiadi Street, Chaoyang District, Beijing, 100102 China; 2 School of Traditional Chinese Medicine & School of Integrated Chinese and Western Medicine, Nanjing University of Chinese Medicine, Nanjing, China; 3 Department of Gastroenterology, Wangjing Hospital, China Academy of Chinese Medical Sciences, Huajiadi Street, Chaoyang District, Beijing, 100102 China; 4 Department of Academic Development, Wangjing Hospital, China Academy of Chinese Medical Sciences, Huajiadi Street, Chaoyang District, Beijing, 100102 China

**Keywords:** Endocrinology, Gastroenterology, Pathogenesis, Risk factors

## Abstract

Chronic gastritis (CG) and osteoporosis (OP) are common and occult diseases in the elderly and the relationship of these two diseases have been increasingly exposed. We aimed to explore the clinical characteristics and shared mechanisms of CG patients combined with OP. In the cross-sectional study, all participants were selected from BEYOND study. The CG patients were included and classified into two groups, namely OP group and non-OP group. Univariable and multivariable logistic regression methods were used to evaluate the influencing factors. Furthermore, CG and OP-related genes were obtained from Gene Expression Omnibus (GEO) database. Differentially expressed genes (DEGs) were identified using the GEO2R tool and the Venny platform. Protein–protein interaction information was obtained by inputting the intersection targets into the STRING database. The PPI network was constructed by Cytoscape v3.6.0 software again, and the key genes were screened out according to the degree value. Gene function enrichment of DEGs was performed by Webgestalt online tool. One hundred and thirty CG patients were finally included in this study. Univariate correlation analysis showed that age, gender, BMI and coffee were the potential influencing factors for the comorbidity (P < 0.05). Multivariate Logistic regression model found that smoking history, serum PTH and serum β-CTX were positively correlated with OP in CG patients, while serum P1NP and eating fruit had an negative relationship with OP in CG patients. In studies of the shared mechanisms, a total of 76 intersection genes were identified between CG and OP, including CD163, CD14, CCR1, CYBB, CXCL10, SIGLEC1, LILRB2, IGSF6, MS4A6A and CCL8 as the core genes. The biological processes closely related to the occurrence and development of CG and OP mainly involved Ferroptosis, Toll-like receptor signaling pathway, Legionellosis and Chemokine signaling pathway. Our study firstly identified the possible associated factors with OP in the patients with CG, and mined the core genes and related pathways that could be used as biomarkers or potential therapeutic targets to reveal the shared mechanisms.

## Introduction

Chronic gastritis (CG) and osteoporosis (OP) are common and occult diseases in the elderly. According to the data of recent epidemiological studies, there has been a decrease in the proportion of Helicobacter pylori-associated gastritis with an increase in the contribution of other etiological factors^1^. The international survey in 2003–2012 found that the prevalence of CG based on endoscopic diagnosis was close to 90%^2^. Meanwhile, OP is a systemic skeletal disease affecting affect up to 50% of postmenopausal women and 20% of men older than 50 years, with high health and economic burden worldwide^3^. With the progress of OP, osteocalcin will continuously lose and the patients will experience pain and spinal deformation. Moreover, severe osteoporotic fractures may occur; some patients have decreased muscle volume and strength, and are prone to falls, leading to an increased risk of fractures and a decline in quality of life.

In recent years, the relationship between CG and OP has received more attention and has been increasingly exposed^4^. Many patients with CG will experience a significant reduction in bone density throughout the body. Some hormone substances secreted by secretory cells of gastric mucosa can regulate bone calcium, which are closely related to the occurrence of OP^5^. The occurrence of CG is mainly due to Helicobacter pylori infection, diet, lifestyle, and so on^6–9^. The risk factors of OP are mainly concentrated in age, diet, and lifestyle, and OP could be caused by some diseases such as gastrointestinal diseases, endocrine diseases, etc.^10,11^. It is a pity that there are few researches to explore the relationship of these two diseases and the related risk factors.

The shared mechanisms of CG and OP are not very clear at present, which would further hamper our investigation of the comorbidity of these two diseases. In any gastrointestinal disease, the response of low circulating leptin to weight loss may be an important factor in reducing bone mass^4^. It is also found that intestinal microorganisms are closely related to the regulation of intestinal permeability and bone health^12^. Consequently, the identified core genes may become a new research focus, and the obtained molecular mechanisms and signaling pathways contribute to the study of the relationship between CG and OP^13^.

In this study, we implemented the cross-sectional study in 10 communities in Beijing, China^14^. We aimed to analyze the clinical characteristics and associated factors for patients with CG complicated with OP. And we enriched the signaling pathways common to both by mining the shared novel genes of CG and OP, revealing their shared mechanisms. To the best of our knowledge, this might be the first study to analyze the clinical characteristics and explore the shared gene signatures between CG and OP through a cross-sectional study and using a systems biology approach.

## Materials and methods

### Cross-sectional studies

#### Study design

All participants were selected from 1540 community residents who completed past medical history inquiries and physical examinations in Chaoyang and Fengtai districts of Beijing, China from November 2017 to July 2018^14^. In the survey, we collected the general information of 1540 community residents, current diseases, bone mineral density, diet, drug use, biological indicators and other relevant data information. The trial was conducted in accordance with the Declaration of Helsinki. Written informed consent was obtained for all material from each participant. This study had been registered on the Chinese Clinical Trial Registry already. (Website: http://www.chictr.org.cn) (Registration Number: ChiCTR-SOC-17013090).

#### Diagnostic criteria

**Figure 1:**
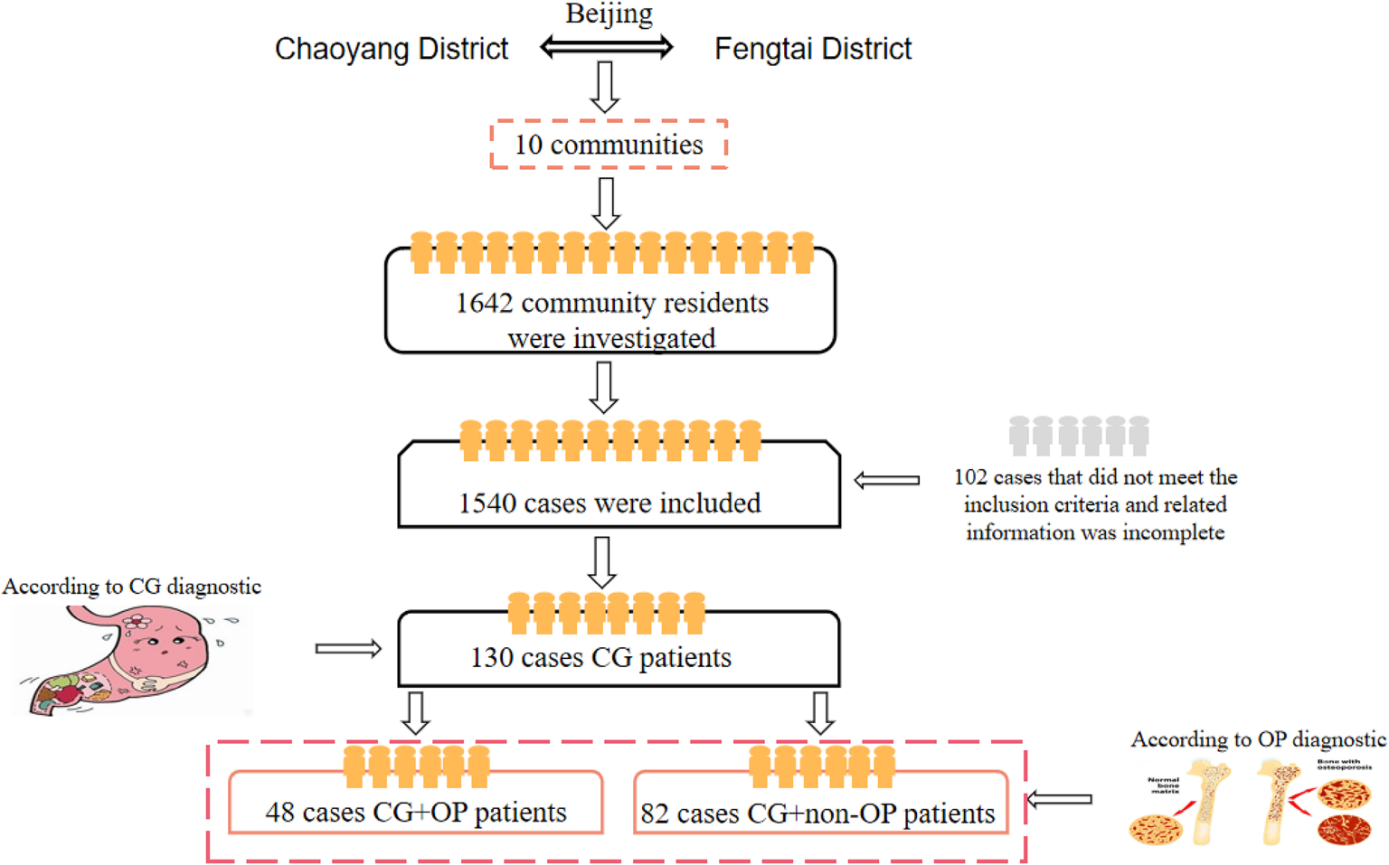
Crowd screening flow chart.

CG patients were determined by clinical diagnosis reports and patient self-report, regardless of the different types of CG^15^. The diagnostic criteria of OP patients referred to the recommendation of the World Health Organization (WHO), namely, taking into account the T value of bone mineral density: T value > − 2.5 SD was non-OP; T value ≤ − 2.5 SD was OP^16^ (Fig. 1).

#### Inclusion and exclusion criteria

The inclusion criteria were as follows: (1) aged from 45 to 80 years; (2) the subjects lived locally lasting for more than 5 years; (3) have clinical diagnosis report and patient self-report diagnosed as CG; (4) the population with complete clinical and laboratory information including BMD, bone metabolic markers.

The exclusion criteria included: (1) the participants who had missing important information; (2) having digestive tract tumors and diseases that affect bone metabolism or calcium absorption, such as diabetes and thyroid diseases, hematological diseases, leukemia, myeloma, systemic lupus erythematosus and kidney diseases; (3) Patients who received drugs or treatments that may affect the study within the first three months of the study, such as glucocorticoids and immunosuppressants.

#### Information acquisition

The study focuses on information collection in three aspects: population characteristics (age, gender, BMI, drinking and smoking), serum biochemical markers (β-CTX, PTH, ALP, P1NP), diet types (fruits, milk, yogurt and coffee).

#### Bone mineral density

Dual-energy X-ray absorptiometry device (Hologic, WI, USA) was used to assess the value of BMD (g/cm^2^). The anteroposterior L1-L4 and left proximal femur including the femoral neck and the total hip BMD were detected, and the T and Z values of each site were recorded. According to the WHO criteria, the OP and non-OP populations were identified based on bone densitometry.

#### Bone metabolism index detection

Fasting blood samples of the participants were collected from 8 a.m. to 9 a.m. in the sitting position. The measurements were conducted through an automated electrochemiluminescence immunoassay system (Roche, Cobas E601, Germany). Additionally, the blood samples were centrifuged to get serum and stored at minus 80 centigrade. As a professional third-party testing organization, Guangzhou KingMed Diagnostics Limited Liability Company was responsible for collecting and testing blood samples.

#### Data collection

All the data, including demographics, clinical characteristics, potential influencing factors, laboratory test and BMD results, were checked by the two independent researchers.

### Studies of shared mechanisms

#### Access to disease genes

Microarrays related to CG and OP were acquired from the GEO database (update time: April 10, 2022, https://www.ncbi.nlm.nih.gov/gene). The keywords “chronic gastritis”, “osteoporosis”, and only those genes from “Homo sapiens” were used as the research targets, analyzed, and discussed in this paper. The gene expression profiling data of CG gastric epithelial tissues were obtained under accession number GSE60427, containing 15 patients with CG and 7 healthy controls, whose level was GPL17077; Gene expression profiling data of peripheral blood of OP, number GSE56116, containing 10 OP patients and 3 healthy controls, whose platform was used as GPL4133.

#### Analysis and selection of DEGs

GEO2R (http://www.ncbi.nlm.nih.gov/geo/geo2r/) is an intelligent online analysis tool that can integrate and analyze two datasets under the same experimental conditions, or split any GEO data^17^. In this study, GEO2R tool was used to analyze the genes between CG and OP. The genes that adjusted the *P* < 0.05 and |log2FC|(Fold Change) > 1 were considered as DEGs. After obtaining the differentially expressed genes, online Venn analysis tool (http://bioinformatics.psb.ugent.be/webtools/Venn/) was used to obtain DEGs intersection and specific DEGs shared by CG and OP.

#### Protein–protein interaction analysis

To identify the relationships among the intersection targets, we used the STRING database (https://string-db.org/). According to the comprehensive analysis of the topological parameters “closeness,” “betweenness,” and “degree”^18,19^. Subsequently, 10 core genes were further screened out by the cytoHubba plug-in of Cytoscape v3.6.0 software^20–22^.

#### GO (gene ontology) functional enrichment and KEGG signaling pathways analysis

Gene Ontology (GO) is a commonly used bioinformatics tool, which can provide relevant information according to the defined characteristics, including comprehensive information on gene function of a single genome product. GO enrichment analysis can explain gene functions from three aspects: molecular function (MF), biological process (BP) and cellular component (CC). Kyoto Encyclopedia of Genes and Genomes (KEGG) is a database that systematically analyzes the metabolic pathways and functions of gene products in cells. In this study, GO and KEGG analyses were performed using WebGestalt (http://www.webgestalt.org/)^23,24^.

### Statistical analysis

The classification variable was expressed as frequency and percentage (%). The χ^2^ test or Fisher exact test or Bonferroni’s method was used to compare the categorical variables between the two groups. Student’s *t* test or Mann–Whitney *U* test was used to analyze continuous variables. In addition, we conducted an in-depth analysis of the clinical characteristics of the OP and non-OP groups. Univariate and multivariate logistic regression models were used to explore the risk factors of CG complicated with OP. All the analysis was carried out with IBM SPSS Statistics 23.0 software. Bidirectional alpha less than 0.05 was considered statistically significant.

### Ethics approval

The study was approved by the Ethics Committee of Wangjing Hospital, Chinese Academy of Chinese Medical Sciences (NO. WJEC-KT-2017-020-P001) and registered on the platform of China Clinical Trial Registry (NO. ChiCTR-SOC-17013090).

### Consent to participate

Informed consent was obtained from all individual participants included in the study.

## Results

### Cross-sectional studies

#### Participants characteristics

**Figure 2:**
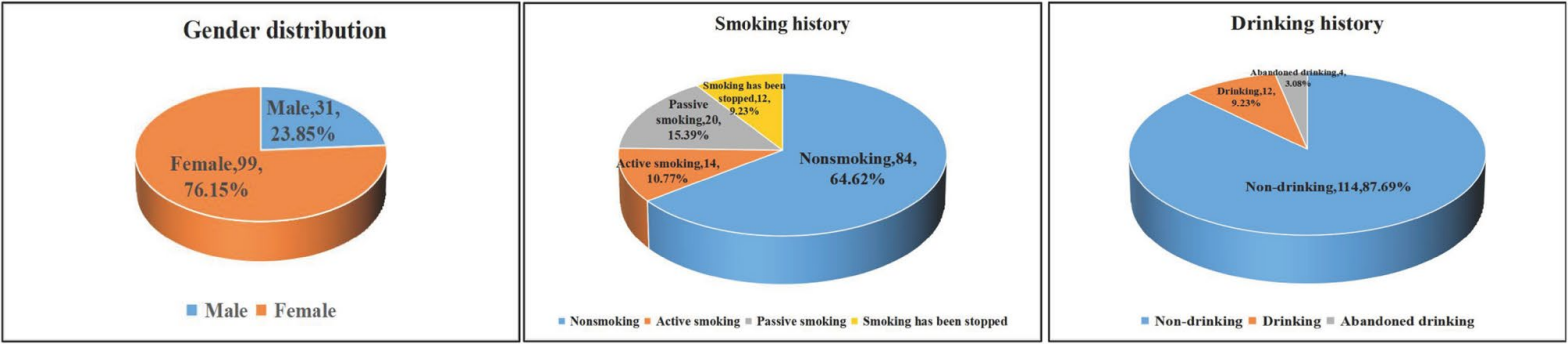
Descriptive characteristics of the study population.

A total of 130 CG patients were included in this study. According to whether CG patients combined with OP, the subjects were divided into OP group (48 cases) and non-OP group (82 cases). Compared with the non-OP group, the patients of OP group were older, including 5 males (10.4%) and 43 females (89.6%) in the OP group, 26 males (31.7%) and 56 females (68.3%) in the non-OP group, and the BMI of patients in OP group was lower than that of the non-OP group. There were significant differences in age (*P* = 0.04), gender (*P* = 0.01), and BMI (*P* = 0.03) between OP and non-OP patients. In addition, there was no statistically significant difference in drinking and smoking history (Table 1, Fig. 2).

**Table 1:** Descriptive characteristics of the study population.

Characteristics	Total (n = 130)	CG + OP (n = 48)	CG + non-OP (n = 82)	*t/2*	*P*
Age	65.11 ± 7.96	66.94 ± 7.73	64.04 ± 7.94	2.05	0.04
Gender					
Male	31 (23.85%)	5 (10.4%)	26 (31.7%)	7.56	0.01
Female	99 (76.15%)	43 (89.6%)	56 (68.3%)		
BMI	24.20 ± 2.97	23.46 ± 2.69	24.63 ± 3.05	− 2.27	0.03
Drinking history					
Non-drinking	114 (87.69%)	42 (87.50%)	72 (87.80%)	0.36	0.84
Drinking	12 (1.54%)	4 (8.30%)	8 (9.80%)		
Abandoned drinking	4 (3.08%)	2 (4.20%)	2 (2.40%)		
Smoking history					
Nonsmoking	84 (64.62%)	32 (66.70%)	52 (63.40%)	3.81	0.28
Active smoking	14 (10.77%)	2 (4.20%)	12 (14.60%)		
Passive smoking	20 (15.39%)	9 (18.80%)	11 (13.40%)		
Smoking has been stopped	12 (9.23%)	5 (10.40%)	7 (8.50%)		

Data are x ± S, n (%), or n/N (%). p values were calculated by the Mann–Whitney *U* test, χ^2^ test, or Fisher’s exact test, as appropriate.

#### Comparison of biochemical markers

There was no significant difference in β-CTX, PTH, ALP, and P1NP between OP and non-OP patients (Table 2).

**Table 2:** Comparison of biochemical markers between two groups.

Group	β-CTX (ng/mL)	PTH (pmol/L)	ALP (U/L)	P1NP (ng/mL)
CG + OP	0.32 ± 0.13	3.50 ± 1.26	78.65 ± 19.38	53.21 ± 21.36
CG + non-OP	0.31 ± 0.13	3.24 ± 2.14	84.15 ± 21.55	58.43 ± 25.13
*t*	0.65	1.24	− 1.86	− 1.59
*P*	0.52	0.22	0.07	0.12

#### Comparison of diet types

**Figure 3:**
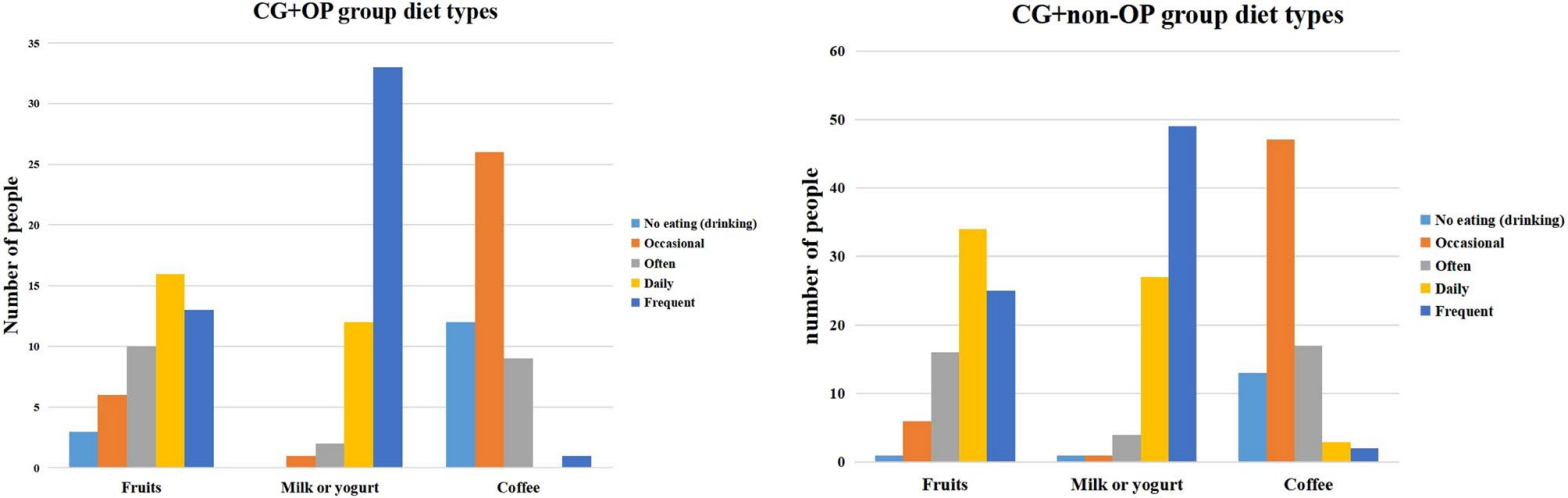
CG + OP and CG + non-OP groups diet types.

In terms of diet types, coffee had a significant difference between OP and non-OP patients, while fruits, milk or yogurt had no significant difference between OP and non-OP patients (Table 3, Fig. 3).

**Table 3:** Comparison of diet types between groups.

Group	CG + OP					CG + non-OP					*F*	*P*
	No eating (drinking)	Occasional	Often	Daily	Frequent	No eating (drinking)	Occasional	Often	Daily	Frequent		
Fruits	3 (6.3%)	6 (12.5%)	10 (20.8%)	16 (33.3%)	13 (27.1%)	1 (1.2%)	6 (7.3%)	16 (19.5%)	34 (41.5%)	25 (30.5%)	2.51	0.12
Milk or yogurt	0 (0.0%)	1 (2.1%)	2 (4.2%)	12 (25.0%)	33 (68.8%)	1 (1.2%)	1 (1.2%)	4 (4.9%)	27 (32.9%)	49 (59.8%)	0.20	0.66
Coffee	12 (25.0%)	26 (54.2%)	9 (18.8%)	0 (0.0%)	1 (2.1%)	13 (15.9%)	47 (57.3%)	17 (20.7%)	3 (3.7%)	2 (2.4%)	4.69	0.03

#### Multivariable logistic regression analysis for influencing factors

**Figure 4:**
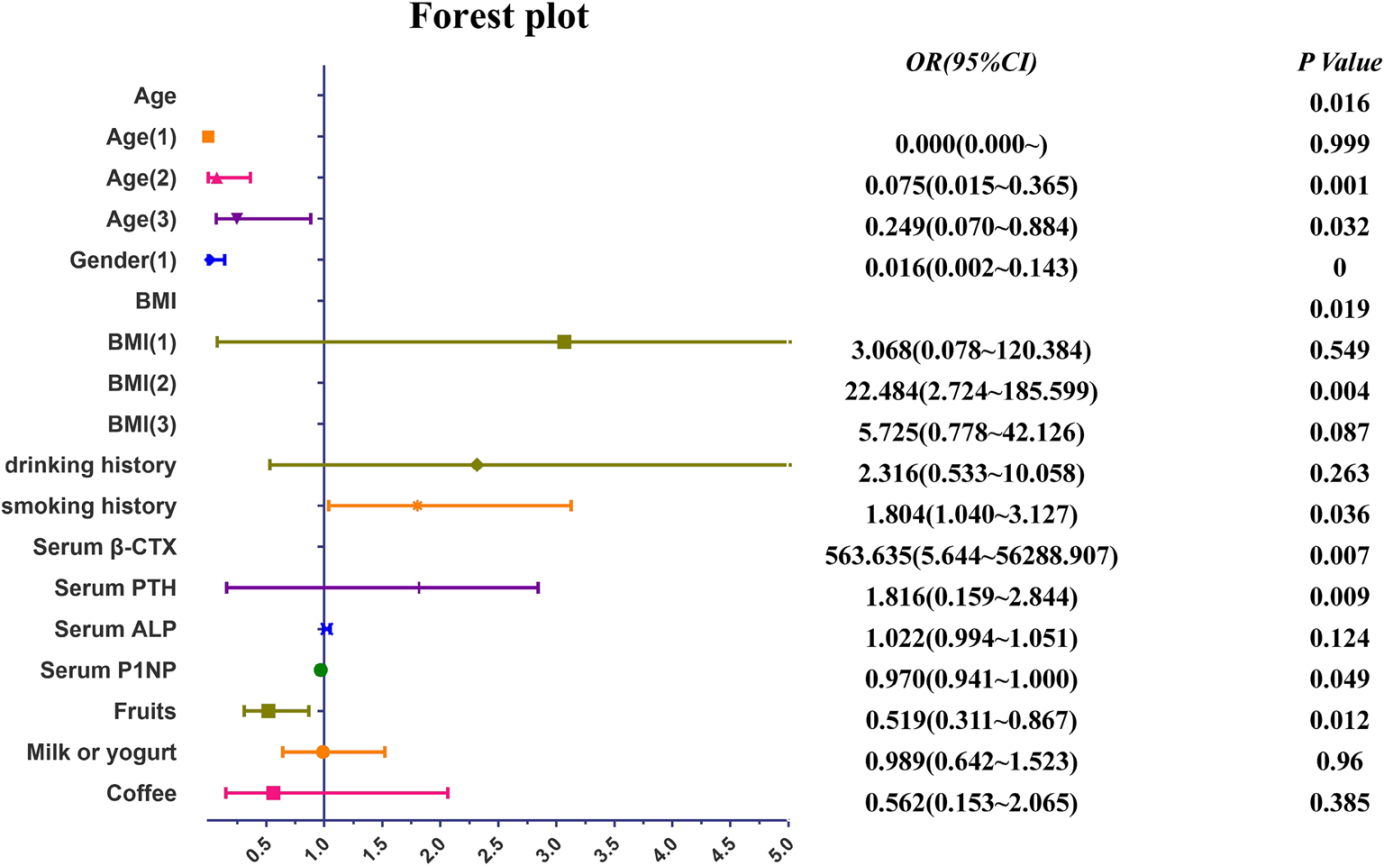
Forest plot of factors associated with OP in CG patients.

Age, gender, and BMI were taken as covariates for classification, and their correlations were taken as independent variables for Logistic regression equation analysis. Resulting that age, gender, BMI, serum PTH, serum β-CTX, serum P1NP and fruit were all related factors for CG combined with OP (Table 4, Fig. 4). Ultimately, smoking history, serum PTH and serum β-CTX were positively correlated with OP in CG patients, while serum P1NP and eating fruit had an obviously negative relationship with OP in CG patients.

**Table 4:** Multivariable logistic regression analysis.

Factor	*β*	Wald	*P*	OR (95% CI)
Age		10.360	0.016	
Age (1)	− 22.207	0.000	0.999	0.000 (0.000 ~)
Age (2)	− 2.597	10.254	0.001	0.075 (0.015 ~ 0.365)
Age (3)	− 1.389	4.624	0.032	0.249 (0.070 ~ 0.884)
Gender (1)	− 4.109	13.817	0.000	0.016 (0.002 ~ 0.143)
BMI		9.959	0.019	
BMI (1)	1.121	0.358	0.549	3.068 (0.078 ~ 120.384)
BMI (2)	3.113	8.354	0.004	22.484 (2.724 ~ 185.599)
BMI (3)	1.745	2.936	0.087	5.725 (0.778 ~ 42.126)
Drinking history	0.840	1.255	0.263	2.316 (0.533 ~ 10.058)
Smoking history	0.590	4.413	0.036	1.804 (1.040 ~ 3.127)
Serum β-CTX	6.334	7.272	0.007	563.635 (5.644 ~ 56,288.907)
Serum PTH	0.596	6.781	0.009	1.816 (0.159 ~ 2.844)
Serum ALP	0.022	2.366	0.124	1.022 (0.994 ~ 1.051)
Serum P1NP	− 0.031	3.889	0.049	0.970 (0.941 ~ 1.000)
Fruits	− 0.655	6.276	0.012	0.519 (0.311 ~ 0.867)
Milk or yogurt	− 0.011	0.002	0.960	0.989 (0.642 ~ 1.523)
Coffee	− 0.576	0.754	0.385	0.562 (0.153 ~ 2.065)

### Studies of comorbid mechanisms

#### Disease genes of OP and CG

**Figure 5:**
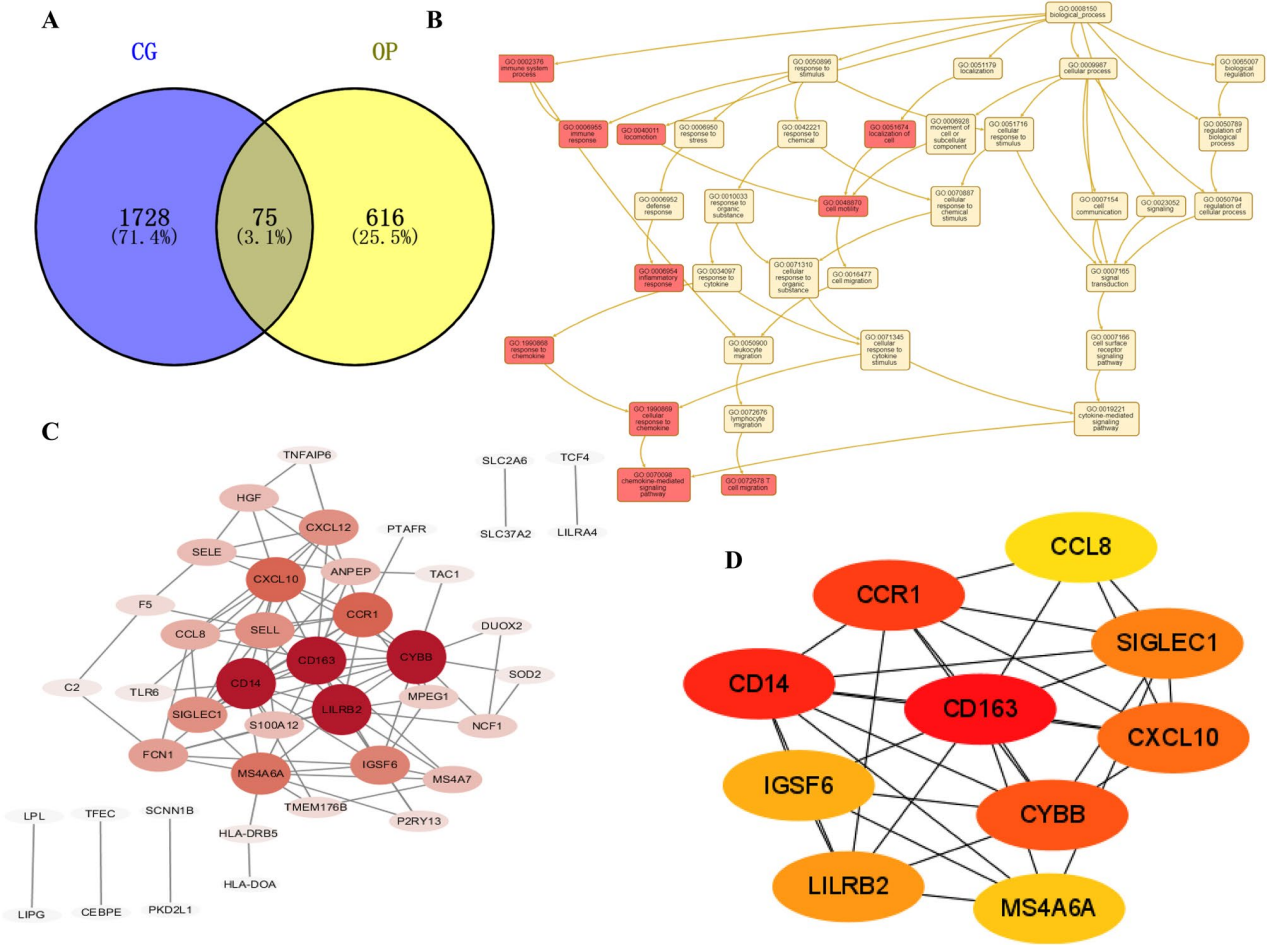

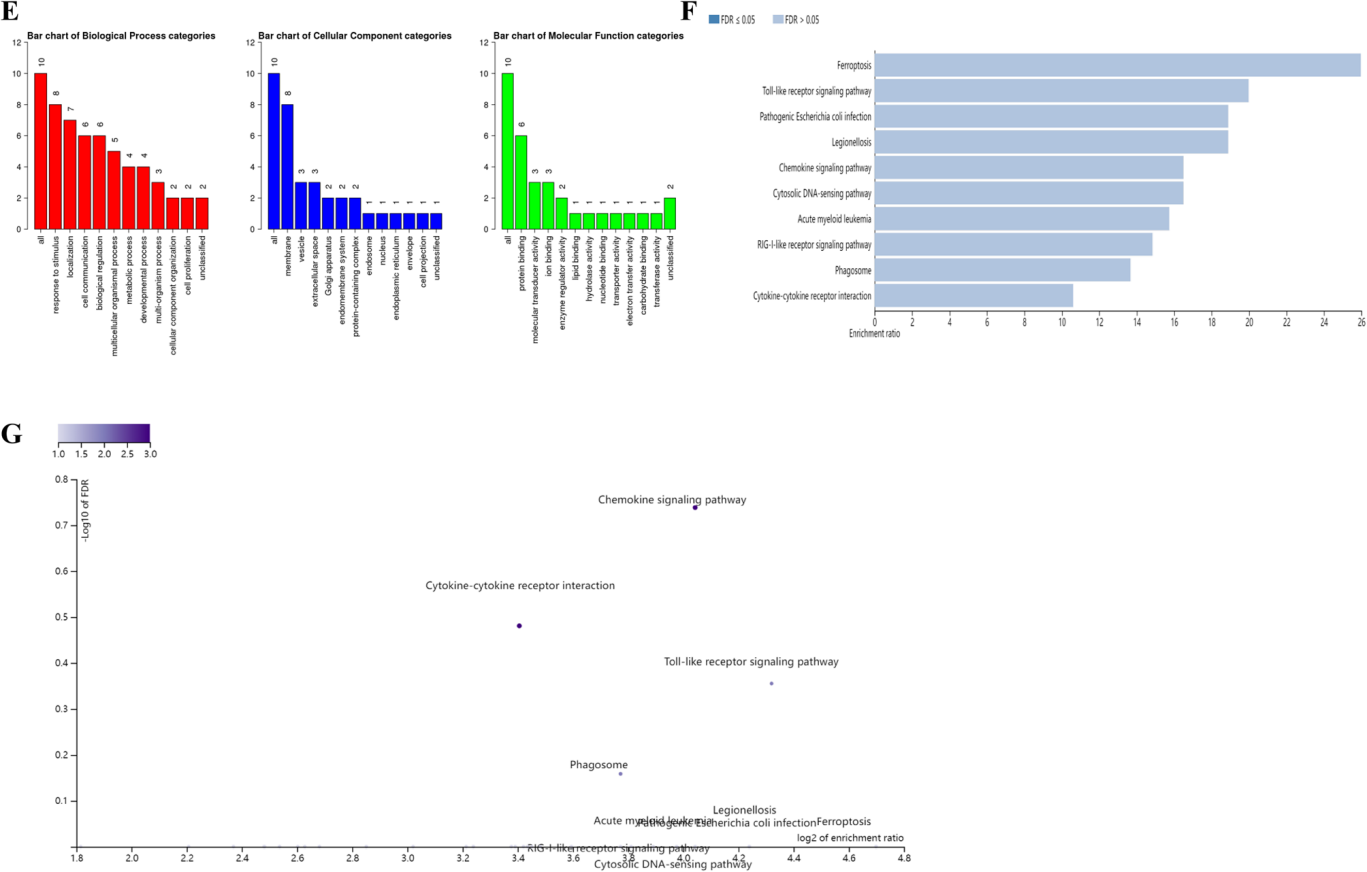
Venn diagram of CG-OP intersection targets (**A**), DAG (**B**), PPI network and topology analysis (**C**,**D**), GO functional enrichment analysis (**E**), KEGG enrichment analysis of signaling pathways (**F**,**G**).

*P* < 0.05 and |log2FC|(Fold Change) > 1 were as criteria for defining differential genes. Through GEO2R analysis, we found 1803 CG DEGs and 691 OP DEGs. Then, the DEGs of CG and OP were analyzed via the Venn platform, and a total of 75 DEGs were obtained. These are presented in Fig. 5A and Table 5.

**Table 5:** Common gene of CG and OP.

Number	Gene	Number	Gene	Number	Gene
1	ANO3	27	ICAM4	53	PTAFR
2	ANPEP	28	IGSF6	54	RANBP17
3	ATP8B3	29	IL19	55	RASSF4
4	C2	30	KCNK12	56	RNF17
5	CADM3	31	KCNQ1	57	S100A12
6	CCL8	32	KCTD15	58	SCG5
7	CCR1	33	KIAA2022	59	SCML4
8	CD14	34	LILRA4	60	SCNN1B
9	CD163	35	LILRB2	61	SELE
10	CEBPE	36	LILRB3	62	SELL
11	CXCL10	37	LIPG	63	SIGLEC1
12	CXCL12	38	LOC100505622	64	SIRPB2
13	CXorf21	39	LPL	65	SLC2A6
14	CYBB	40	MPEG1	66	SLC37A2
15	ANO3	41	MRAS	67	SLC6A12
16	CYP1B1-AS1	42	MS4A6A	68	SNX29
17	DRD3	43	MS4A7	69	SOD2
18	DUOX2	44	NAIP	70	TAC1
19	ESR2	45	NCF1	71	TCF4
20	F5	46	NPCDR1	72	TFEC
21	FCN1	47	OLFM1	73	TLR6
22	FSCN1	48	OLIG1	74	TMEM176B
23	HDAC9	49	P2RY13	75	TNFAIP6
24	HGF	50	PKD2L1		
25	HLA-DOA	51	PLEKHA2		
26	HLA-DRB5	52	PPP1R1C		

#### PPI network construction and core gene screening

A total of 10 core genes were obtained by a 12-parameter correlation analysis of 75 intersection genes with cytoHubba. Having thus obtained the PPI data, we imported these data into Cytoscape to plot the PPI network. These are presented in Fig. 5B–D and Table 6.

**Table 6:** Core gene of CG and OP.

Number	Gene	Number	Gene
1	CD163	6	SIGLEC1
2	CD14	7	LILRB2
3	CCR1	8	IGSF6
4	CYBB	9	MS4A6A
5	CXCL10	10	CCL8

#### GO enrichment analysis and KEGG pathways enrichment analysis

The results of GO enrichment analysis consist of biological processes (BP), cell components (CC), and molecular functions (MF). To further study the biological function of key targets, an enrichment analysis of KEGG pathway was carried out. Meanwhile, the top 10 signaling pathways of key targets, including Ferroptosis, Toll-like receptor signaling pathway, Pathogenic Escherichia coli infection, Legionellosis, Chemokine signaling pathway, Cytosolic DNA-sensing pathway, Acute myeloid leukemia, RIG-I-like receptor signaling pathway, Phagosome, and Cytokine-cytokine receptor interaction (Fig. 5E–G).

## Discussion

CG and OP are highly prevalent diseases around the world. A previous study showed that OP could be caused by gastrointestinal diseases, endocrine diseases, etc.^10,11^. With the progressive increase in the prevalence of CG and OP, it is necessary to explore the clinical characteristics and mechanism in these 2 diseases and discover the associated factors and early potential targets to prevent disease development^25,26^.

Helicobacter pylori (Hp) infection seemed to be one of the most important factors that may increase the risk of CG patients complicated with OP. A Japanese research of 230 male patients aged 50–60 years found that HP infection increased the risk of low bone mass by 1.83 times and atrophic gastritis increased the risk of low bone mass by 2.22 times. Therefore, HP infection and atrophic gastritis were considered as high-risk factors for OP^27^. However, the relationship between HP infection and OP is also controversial. Through a cross-sectional study of 85 female patients, Brazilian researchers found that HP-associated gastritis and autoimmune gastritis were not risk factors for abnormal bone mass^28^. Iranian researchers included 967 elderly people over 60 years old. The results also showed that HP infection was not significantly associated with bone mineral density change^29^. The prevalence of CG increases with age^30^. This is mainly related to the increase in Hp infection rate with age, and atrophy, intestinal metaplasia and “aging” are also related to a certain extent. In our study, CG patients with OP were older than those without OP. In addition, the high risk of elderly patients with OP may be attributed to their overall poor health, bone loss accelerated and an increase in the number of complications.

In addition, our study found that there were gender differences in patients with CG complicated with OP. Marked gender differences in the disease have been confirmed in other observational studies reporting the total incidence of autoimmune gastritis in the population is 2%, in which the incidence of elderly women is as high as 4% to 5%, and there is no race or region specificity^31^.

Poor lifestyle is a commonly associated factor for the occurrence of CG patients complicated with OP, including dietary preferences and drink types. A retrospective study conducted in Shanghai, China found that eating spicy food and eating fast food were risk factors for the progress of chronic atrophic gastritis^7^ A longitudinal study of 2682 cases in the Taiwan region of China also confirmed that the T score of bone mineral density in people with medium and high intake of coffee was higher, so it was inferred that coffee intake was negatively correlated with the risk of osteoporosis^32^. Moreover, smoking can lead to many health problems. Cigarettes contain many chemical components, which have great harm to the human body, especially when they are burned. One component of cigarettes is cadmium, and smoking is the main source of cadmium exposure for smokers^33^. Recent studies have shown that even low levels of exposure to cadmium increase the risk of osteoporosis and fractures^34,35^. This is consistent with our findings. We found that long-term smoking in CG patients is prone to OP, but our study found that coffee is not statistically significant, which may be related to our small sample size. However, in this study, poor eating habits are also common risk factors for OP in CG patients. Various trace nutrients in fruits are conducive to reducing the risk of chronic diseases^36^. Fruit intake was positively correlated with bone mineral density and negatively correlated with fracture risk^11,37–39^. In this study, the daily diet survey of the CG population found that CG patients who did not guarantee daily intake of fruits had a higher risk of OP.

Parathyroid hormone (PTH) can finely regulate bone synthesis and catabolism, and play an important role in the differentiation, maturation and apoptosis of osteoblasts and osteoclasts. PTH can up-regulate the expression of receptor activator of NF-κB ligand (RANKL) through parathyroid hormone receptor signaling pathway, thereby inducing bone resorption^40^. Studies have shown that PTH has dual effects on bone formation and bone resorption. The biological effect of PTH depends on its dose. Under the action of continuous high-dose PTH, osteoclast activity exceeds osteoblasts, resulting in bone loss greater than bone formation. Intermittent low-dose PTH promotes bone metabolism, and bone formation is greater than bone resorption, which can play a role in promoting bone formation^41^. Recent studies have reported that PTH can stimulate gastrin secretion and affect the absorption of Ca and P^42–45^, inducing OP, which was in line with our study. The International Osteoporosis Foundation (IOF) and the International Federation of Clinical Chemistry (IFCC) Working Group on the Standardization of Bone Markers recommended type β-I collagen carboxy-terminal peptide (β-CTX) and type I procollagen amino-terminal peptide (P1NP) as bone turnover markers (BTMs). β-CTX is a type I collagen degradation product. When the osteoclast activity increases, a large number of type I collagen degradation will occur, resulting in an increase in the level of β-CTX. Therefore, the bone formation and bone absorption in postmenopausal women are uncoupled, and the serum β-CTX level increases^46^. PINP is produced in the process of type I collagen synthesis. The content of PINP in serum reflects the ability of osteoblasts to synthesize bone collagen, and can be used to monitor the vitality of osteoblasts and bone formation. Many studies have confirmed that among numerous bone metabolism indicators, PINP has high specificity and sensitivity in predicting the occurrence of osteoporosis, evaluating bone mass, and monitoring the efficacy of anti-osteoporosis^47^. Chen et al. found that PINP was the best predictor of BMD increase after treatment in postmenopausal women with osteoporosis^48^. It was also found that the serum PINP levels in both male and female patients with osteoporosis were significantly lower than those in the normal group, and were positively correlated with BMD of the femoral neck, indicating that the osteoblast function in patients with osteoporosis decreased, the synthesis of collagen decreased, and bone formation decreased^49^. Our study also yielded the same results. CG people have with higher PTH and serum β-CTX, as well as lower serum P1NP have a higher risk of OP.

In studies of shared mechanisms, a total of 75 intersection genes were obtained and topologically analyzed by bioinformatic analysis of the two microarrays containing CG gastric epithelial tissues expression microarray GSE60427 and OP serum gene expression microarray GSE56116 from the GEO database, and the results indicated that CD163, CD14, CCR1, CYBB, CXCL10, SIGLEC1, LILRB2, IGSF6, MS4A6A, and CCL8 might be key targets for the treatment of CG with OP. Related studies have found that CD163 and CD14 are increased in H. pylori infection, especially in patients with peptic ulcers. CD163 and CD14 are positively associated with the severity of H. pylori infection and CD163 was also positively correlated with fracture^50,51^. Soluble CD14(sCD14), a proinflammatory cytokine, is primarily derived from macrophages/monocytes that can differentiate into osteoclasts, and sCD14 is an inflammatory marker associated with osteoclasts^52^. Gastritis and gastric tumorigenesis are associated with upregulation of CCR1^53^. Related studies have shown that overexpression of CCR1 will increase the number of osteoclasts and osteoclast size^54^. CYBB is highly expressed in gastric cancer, and CYBB was identified as potential biomarkers in gastric cancer^55^. CG people produced higher levels of CXCL10 than healthy people^56^. Studies have found that CXCL10 is closely related to osteoclasts, and its downregulation can inhibit osteoclastogenesis^57^. SIGLEC1 provides vital pro-anabolic support to osteoblasts during both bone homeostasis and repair^58^. Correlative studies confirmed that LILRB2 is expressed on cultured osteoclast precursor cells derived from peripheral blood mononuclear cells and may inhibit osteoclast development^59^. IGSF6 has strong links to CG as a positional and functional candidate for susceptibility to inflammatory bowel disease (IBD)^60^. Relevant studies also found that MS4A6A was closely related to esophageal cancer and ovarian cancer and was its biomarker^61^, so we speculated that it also had a close correlation with the occurrence of CG and OP. CCL8 expressed and upregulated in spinal neurons after colonic inflammation, are involved in the maintenance of visceral hyperalgesia via the activation of spinal ERK^62^. And CCL8 is abundant in synovium and is associated with erosion of cartilage^63^.

Our results revealed that Ferroptosis, Toll-like receptor signaling pathway, and so on were the major signaling pathways shared by CG and OP. Ni et al. found that Ferroptosis can attenuate gastric cancer cell stemness^64^. Ferroptosis has a close relationship with both osteoclasts and osteoblasts^65^. Studies have reported that disorders of iron metabolism, including iron deficiency and iron overload, can lead to osteoporosis^66,67^. Song et al. found that FA complementation group D2 (FANCD2) suppresses erastin-induced ferroptosis in bone mesenchymal stem cells (BMSCs), and FANCD2 reduces iron accumulation and lipid peroxidation in ferroptosis^68^. Toll-like receptors (TLRs) are innate immune cell receptors. Helicobacter pylori infection can stimulate innate immune response through toll-like receptor (TLR) and nuclear factor-κB activation induces the COX-2/PGE2 pathway, which leads to gastritis and gastric cancer^69^. And osteoblasts express TLR2, activation of TLR2 in RANKL stimulates bone marrow macrophages (BMMs) resulting in inhibition of osteoclast differentiation and formation^70^. Helicobacter pylori is a Gram-negative bacterium belonging to the epsilon-proteobacteria class, *H*. *pylori* inhabits the stomach of half of the human population worldwide, and infected individuals suffer from chronic gastritis^71^. Dysbiosis of the gut microbiota can contribute to the development and progression of osteoporosis by regulating body immunity, hormone levels, as well as bone metabolism, and is an important cause of osteoporosis in elderly women^72^. Legionella species are a large collection of environmental Gram-negative bacteria that have evolved the capacity to replicate to high numbers in a range of eukaryotic cells. This trait enables some Legionella to be pathogenic to humans, particularly when the individual is immunocompromised^73^. However, there are few studies on Legionella on CG and OP. H pylori promoted gastric epithelial cell senescence in vitro and in vivo in a manner that depended on C-X-C motif chemokine receptor 2 (CXCR2) signaling, and inhibition of CXCR2 signaling is suggested as a potential preventive therapy for targeting H pylori-induced atrophic gastritis^74^.

The major strength of this report lies in practical value for the management of patients with CG complicated with OP and provides some references for future researches. We are further optimizing our questionnaire and program, and want to further collect national population data, in order to provide better evidence. In studies of shared mechanisms, the cross parts of the disease genes network of CG and OP obtained in this study are the most notable genes, which is also a breakthrough point in the relationship between CG and OP. At the same time, it can also provide a more reliable path and potential targets for drugs to intervene in two interrelated diseases simultaneously. However, due to the limitation of screening conditions, only the main genes, targets and signaling pathways can be analyzed, which makes the results limited to a certain extent. Secondly, the integration of different genes depends on the development of bioinformatics technologies. The integrity and accuracy of the disease database directly determine the reliability of the integrated information. Therefore, further validations and supports of experiments in vivo and in vitro are necessary for follow-up researches.

Despite the importance of the aforementioned findings, the current study, however, has some limitations. Firstly, the diagnosis of CG is only dependent, and the diagnosis results of pathology and gastroscopy are not tracked, and we have not classified the CG population. Secondly, the scope of this study is limited to Beijing, so that the relatively small sample size might influence the interpretation of our findings. Finally, many details are still not considered fully, such as the study of comorbidities in Traditional Chinese Medicine syndrome^75,76^. So more associated factors of the two diseases still need to be further explored (Supplementary Information).

## Conclusion

Our study reveals that age, gender, BMI, smoking history, PTH, β-CTX, P1NP and intake of fruit may be relevant risk factors for CG and OP, and uncovers the potential shared mechanism of CG and OP by identifying core genes that could be used as biomarkers or as potential therapeutic targets and related pathways through data mining.

## Supplementary Information

Supplementary Information.

## Data Availability

All data used to support the findings of this study are included within the article.
