# Supplementary material for: Clinical features and shared mechanisms of chronic gastritis and osteoporosis
Source: Sci Rep. 2023 Mar 27;13:4991. doi: 10.1038/s41598-023-31541-8 (PMC10042850; doi:10.1038/s41598-023-31541-8)
Supplement: Supplementary file 1 — Supplementary Information. [file 41598_2023_31541_MOESM1_ESM.docx]

**List of communities covered in this study.**

Xibahe Dongli Community, Chaoyang District, Beijing

Liufang Nanli Community, Chaoyang District, Beijing

Laiguangying Community, Chaoyang District, Beijing

Dujiakan Community, Fengtai District, Beijing

Ao Ti Community, Fengtai District, Beijing

Zhujiang Yujing Community, Fengtai District, Beijing

Taiziyu Community, Fengtai District, Beijing

Zhangjiamun Community, Fengtai District, Beijing

Lijiamun Community, Fengtai District, Beijing

Xinyuan Community, Fengtai District, Beijing
